# Gamma Knife treatment of papillary craniopharyngioma: a single-center study of tumor control and predictors of progression

**DOI:** 10.1186/s13014-026-02875-0

**Published:** 2026-07-08

**Authors:** Yi Lin, Kuanyu Wang, Bo Zhang, Dezhi Gao, Guanyun Zhao, Zhixiong Lin, Shibin Sun

**Affiliations:** 1 Department of Neurosurgery, Sanbo Brain Hospital, Capital Medical University, Beijing, China; 2 Department of Stereotactic Radiosurgery, Beijing Tiantan Hospital, Capital Medical University, Beijing, China

**Keywords:** Papillary craniopharyngioma, Gamma Knife radiosurgery, Stereotactic radiosurgery, Progression-free survival, BRAF V600E, Sellar tumor

## Abstract

**Background:**

Papillary craniopharyngioma (PCP) is a rare sellar tumor distinct from adamantinomatous type. Although Gamma Knife radiosurgery (GKRS) treats craniopharyngiomas effectively, dedicated PCP-only GKRS outcome series are scarce. We evaluated tumor control and prognostic factors following GKRS for PCP.

**Methods:**

We retrospectively analyzed 34 patients with histologically confirmed PCP who underwent GKRS at Beijing Tiantan Hospital (2002–2025) for residual/recurrent disease after surgery. Primary endpoint was progression-free survival (PFS). Kaplan-Meier and Cox regression analyses identified prognostic factors.

**Results:**

Thirty-four patients (18 males, mean age 41.7 ± 13.6 years) received GKRS with median marginal dose 12 Gy to median tumor volume 1.10 cm³. With a median clinical follow-up of 54.10 months, the 1-, 3-, and 5-year PFS rates were 91.18%, 82.35%, and 73.53%, respectively. Overall survival was 94.12%. Disease control rate was 70.59%. Exploratory analysis with limited statistical power (10 events, EPV = 3.3) suggested potential associations between tumor volume > 1.1 cm³ (HR = 3.86, *P* = 0.061) and preoperative hypogonadism (HR = 3.94, *P* = 0.058) with progression, though these findings require validation in larger cohorts. Visual deterioration was significantly higher in progression group (80.0% vs. 16.7%, *P* = 0.002). Most patients (61.8%) maintained good quality of life.

**Conclusion:**

GKRS provides effective long-term tumor control for postoperative PCP patients with acceptable safety profile. Exploratory hypothesis-generating analysis suggested potential associations between tumor volume and preoperative gonadal function status with progression, though these findings require validation in adequately powered studies. These findings support GKRS as a valuable treatment option in the multimodal management of PCP, particularly for patients with residual or recurrent disease after surgery.

**Supplementary Information:**

The online version contains supplementary material available at https://doi.org/10.1186/s13014-026-02875-0.

## Background

Craniopharyngioma (CP) is a rare type of brain tumor, accounting for approximately 1.2–4.6% of intracranial tumors [1, 2]. Despite its histological benignity, CP exhibits invasive growth and has strong adhesion to adjacent structures, often leading to symptoms such as visual deficits, endocrine dysfunction, cognitive impairments, and personality changes. Historically, based on histological examination criteria, CP have been classified into two subtypes: adamantinomatous craniopharyngioma (ACP) and papillary craniopharyngioma (PCP). However, due to significant differences between the two subtypes in terms of patient population, imaging characteristics, histopathological features, driver mutations (CTNNB1 vs. BRAF V600E), biological behavior (invasive growth vs. chronic progression), and treatment response (inflammatory pathway dependence vs. targeted drug sensitivity) [3–10], ACP and PCP are now classified as distinct tumor types [7].

The treatment strategy for CP has been the subject of considerable controversy. The traditional view holds that surgical resection is the ideal treatment, but complete resection often carries a significant risk of neuroendocrine complications [11, 12]. In recent years, a multimodal treatment strategy combining subtotal resection with postoperative radiotherapy has gradually gained acceptance, especially for complex cases involving invasion of critical structures [13].

Gamma Knife radiosurgery (GKRS) is a stereotactic radiosurgery technique that delivers high-dose radiation to the tumor target area in a single treatment while maximizing protection of surrounding healthy tissues [14]. GKRS has been widely used in the treatment of CP, particularly in cases of residual or recurrent tumors following surgery [15, 16]. Previous literature reports that the 5-year local control rate for GKRS of CP ranges from 60% to 93% [15–22], but there are very few published papers that explicitly discuss PCP types from a clinical-pathological or surgical perspective. Most of this information remains hidden in articles and meta-analyses focused on adult CP [23]. In these studies, the results of PCP and ACP are generally combined, yet PCP and ACP are two distinct diseases that may exhibit differences in treatment response [24].

Given that most published Gamma Knife radiosurgery (GKRS) series for craniopharyngioma pool adamantinomatous and papillary subtypes, and that PCP-specific outcomes are rarely reported separately, we conducted a dedicated single-center analysis focusing exclusively on histologically confirmed papillary craniopharyngioma treated with GKRS.

Accordingly, this study aims to systematically evaluate tumor control, progression-related risk factors, and treatment-related complications of GKRS for PCP through long-term follow-up of 34 patients, with the goal of informing individualized treatment strategies.

## Methods

### Patient selection

**Fig. 1:**
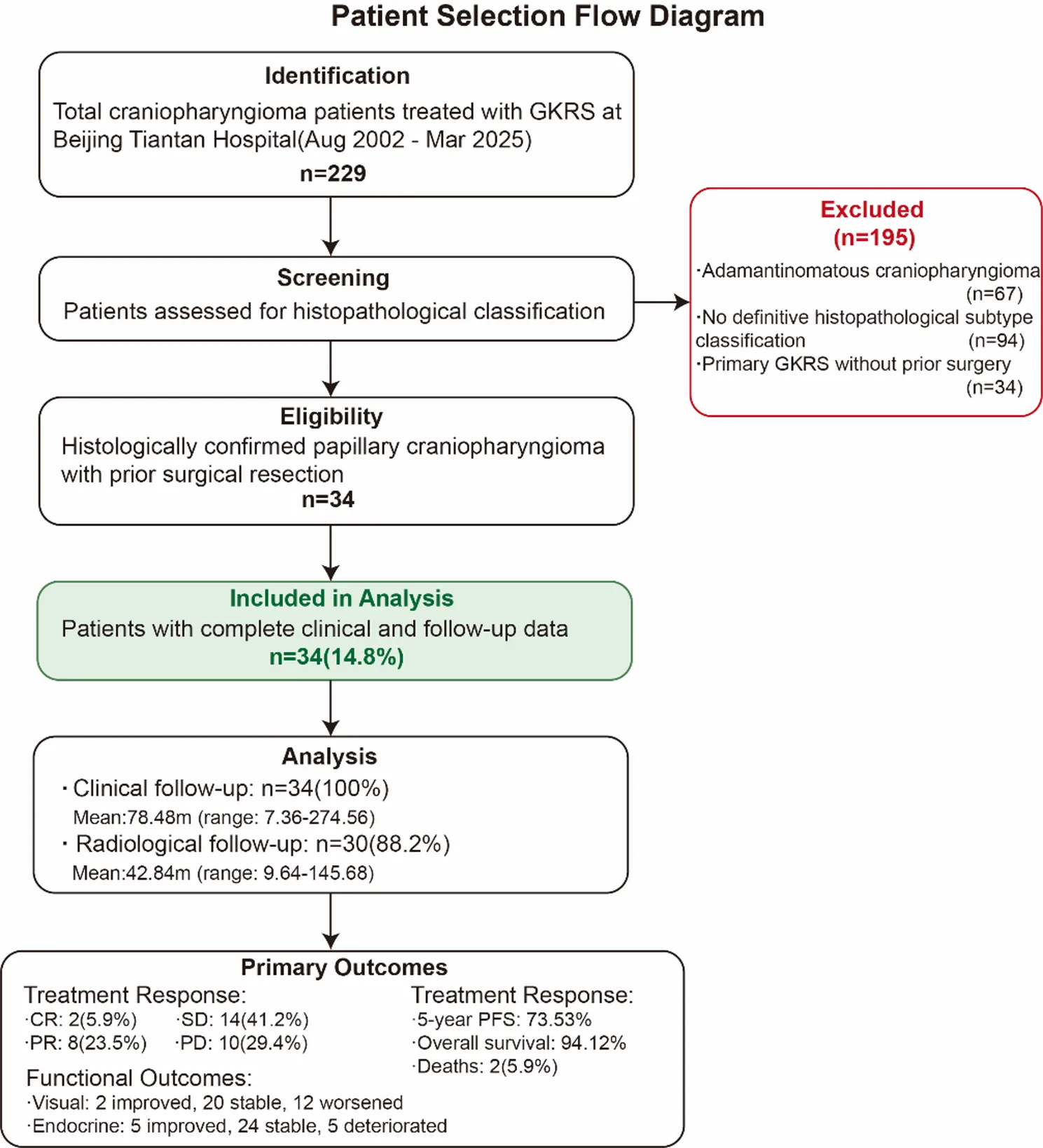
Flow diagram of patient selection and study outcomes

A retrospective study was conducted to evaluate the efficacy and influencing factors of GKRS for PCP treated at the Gamma Knife Center of Beijing Tiantan Hospital, Capital Medical University, from August 2002 to March 2025. Inclusion criteria included histologically confirmed PCP and adequate clinical and radiological follow-up data available for assessment of treatment response and outcomes. Patients without definitive pathological diagnosis, those who received GKRS without prior surgical history, or those with insufficient follow-up data were excluded from the study. Among 229 craniopharyngioma patients who received GKRS during the study period, 34 patients with histologically confirmed PCP met the inclusion criteria and were included in the final analysis (Fig. 1). Treatment Fractionation Approach: In selected cases where tumor geometry and proximity to critical structures precluded safe delivery of adequate prescription dose in a single session, volume-staged radiosurgery was employed. In this approach, the tumor volume was divided into two spatially distinct target regions, with separate GKRS sessions (typically separated by 1–3 months) treating different portions of the tumor to maintain safe dose constraints to adjacent critical structures. Specifically, 4 patients (11.8) underwent planned volume-staged treatment: the initial session targeted tumor regions with greater distance from dose-limiting structures, while the subsequent planned session addressed residual tumor in closer proximity to the optic chiasm or other critical anatomy. For analytical purposes, these two planned sessions were considered a single therapeutic event, as both were part of the initial treatment strategy rather than salvage therapy for tumor progression. Conversely, re-irradiation performed after radiological or clinical evidence of tumor progression was defined as a new treatment event. Residual tumor was defined as visible enhancing tumor on the first post-surgical MRI performed within 3 months after surgery, without interval tumor growth, with GKRS performed as planned adjuvant therapy within 6 months of surgery based on surgical findings and post-surgical imaging. Recurrent tumor was defined as radiographic tumor growth on follow-up MRI compared to immediate post-surgical baseline imaging (≥ 20% volume increase or new enhancing lesion), or MRI evidence of tumor at a site previously documented as complete resection, or interval between surgery and GKRS exceeding 6 months with documented tumor progression on surveillance imaging. Clinical and radiological data were collected from the hospital’s electronic medical record system and the Gamma Knife Center database. This study was approved by the Institutional Review Board of Beijing Tiantan Hospital. Given the retrospective nature of the study, informed consent was waived, and the analysis utilized anonymized clinical data.

### GKRS technique

The Leksell stereotactic frame was secured to each patient’s skull under local anesthesia. High-resolution, contrast-enhanced magnetic resonance imaging (MRI) with 2-mm slice thickness was acquired for treatment planning. Patients received single-session radiosurgery (SRS) using the Leksell Gamma Knife Model C (Elekta AB) from January 2007 to October 2011, after which the system was upgraded to the Leksell Gamma Knife Perfexion (Elekta AB). In 2021, the system was further upgraded to the Leksell Gamma Knife Icon (Elekta AB), which incorporates cone-beam computed tomography (CBCT) image guidance for enhanced precision in adaptive stereotactic radiosurgery. Treatment planning was performed using the GammaPlan system (Elekta AB).

Target definition: The treatment target encompassed the entire visible tumor on T1-weighted contrast-enhanced MRI, including both solid and cystic components. Given the small tumor volumes in our cohort (median 1.10 cm³), cyst walls and non-enhancing cyst fluid were included when integrated with the tumor mass. Target delineation was performed directly on MRI without margin expansions, as the stereotactic frame provides submillimeter positioning accuracy.

Dose prescription and constraints: Prescription dose and isodose line selection (typically 50–55%) were determined based on tumor volume and proximity to critical structures. Maximum point dose to optic apparatus was constrained to < 9 Gy across all eras, with evolving technological improvements enabling better conformality: Perfexion (2011–2021) introduced sectored collimation for improved dose shaping compared to Model C (2002–2011), while Icon (2021–2025) further enhanced conformality through refined collimator design and cone-beam CT guidance. These upgrades improved shot size options and dose fall-off gradients at the tumor-optic interface, providing better visual structure protection. Other critical structures (hypothalamus, pituitary stalk, brainstem) were constrained based on established tolerance doses.

Fractionated radiosurgery: Four patients (11.8%) underwent planned fractionated radiosurgery when optic proximity precluded safe single-session delivery. This differs from spatial volume-staging by providing complete tumor coverage per session while distributing dose temporally. Both sessions were considered a single therapeutic event for analysis.

### Collected variables

Preoperative variables included age at onset, gender, presence of calcification, tumor characteristics, preoperative endocrine comorbidities, Karnofsky Performance Status (KPS) score, surgical resection approach, indication for GKRS treatment, number of recurrences in recurrent patients, and time from surgery to recurrence in patients with recurrent disease. Treatment variables encompassed age at GKRS, tumor volume, central dose, margin dose, isodose line, and maximum dose to adjacent critical structures. Outcome variables included visual function, endocrine function, quality of life, and radiological tumor response. Detailed definitions of outcome assessments are provided in Sect.  4.

### Outcome definitions

#### Visual outcomes

Due to variations in follow-up institutions over the 23-year study period, standardized ophthalmological assessment protocols were not consistently available. Therefore, visual outcomes after GKRS were classified based on patients’ subjective reports during clinical follow-up combined with ophthalmological examination results when available. Patients were categorized as having improved, stable, or deteriorated vision compared to their pre-GKRS baseline.

#### Endocrine outcomes

Endocrine outcomes were evaluated based on changes in hormone replacement therapy documented during follow-up, either at our institution or reported from local hospitals. Given the heterogeneity in follow-up protocols and laboratory assessments across different centers, endocrine status was inferred indirectly through therapeutic adjustments. Deterioration was defined as: (1) initiation of new hormone replacement therapy for previously unaffected axes, (2) ≥ 50% increase in existing hormone replacement dosages sustained for at least 3 months, or (3) addition of a new hormone replacement medication to manage inadequately controlled symptoms. Unchanged dosages without new symptoms were considered stable, while reduced dosages with effective symptom control were regarded as improvement.

#### Quality of life

Quality of life was retrospectively assessed based on available medical records and categorized into three grades. Mild impact was characterized by partial visual field defects, endocrine alterations, or mild obesity that did not affect daily activities and allowed maintenance of normal life. Moderate impact included monocular blindness that did not interfere with daily activities, endocrine disorders or other special conditions, or intellectual, emotional, or visual problems requiring home observation or retirement, possibly accompanied by depression. Severe impact was defined as bilateral blindness or severe bilateral visual field defects with low vision that affected normal life or resulted in inability to perform activities of daily living.

#### Radiological response

Tumor response on follow-up MRI was categorized using volumetric criteria adapted from Response Evaluation Criteria in Solid Tumors (RECIST) principles for three-dimensional tumor assessment [25]. Response assessment was based on the best radiological response observed during the follow-up period, defined as the maximum tumor shrinkage (or minimum growth) documented on serial MRI examinations compared to the pre-GKRS baseline volume. Complete response (CR) was defined as tumor disappearance. Partial response (PR) was defined as tumor volume reduction ≥ 30%. Stable disease (SD) was defined as volume change between − 30% and + 20%. Progressive disease (PD) was defined as volume increase > 20%. The objective response rate (ORR) was calculated as the proportion of patients achieving CR or PR. The disease control rate (DCR) was calculated as the proportion of patients achieving CR, PR, or SD.

Progression-free survival (PFS) was defined as the time from GKRS to radiological progression or death, whichever occurred first. Radiological progression was defined as tumor volume increase > 20% on MRI compared to the smallest post-GKRS volume (corresponding to progressive disease [PD] in our four-category response assessment), or new enhancing tumor at a previously treated site. Clinical symptoms alone without radiological confirmation were not considered progression events for PFS analysis. Patients without radiological progression or death were censored at the date of last radiological follow-up.

### Statistical analysis

Normality of continuous variables was assessed using the Shapiro-Wilk test. Normally distributed continuous variables are presented as mean ± standard deviation (SD) and were compared using two-tailed Student’s t-tests. Non-normally distributed continuous variables are expressed as median with interquartile range (IQR) and were compared using the Mann-Whitney U test. Categorical variables are presented as frequencies and percentages and were compared using the chi-square test or Fisher’s exact test when more than 20% of expected cell frequencies were less than 5. Progression-free survival (PFS) was estimated using the Kaplan-Meier method, and survival curves were compared using the log-rank test. For stratified survival analysis, continuous variables were dichotomized at the median value as a pre-specified cut-point to avoid overfitting bias associated with data-driven threshold selection in small samples. Cox proportional hazards regression models were employed to explore associations between potential prognostic factors and PFS. Given the limited number of progression events (*N* = 10) relative to candidate predictors, we employed a clinically guided pre-specified multivariable model rather than data-driven stepwise selection procedures to minimize overfitting and model instability. Variables entered into the multivariable model were selected based on clinical relevance and univariable association (*P* < 0.2), including tumor volume (as a continuous variable and dichotomized at median), preoperative hypogonadism, and preoperative KPS score. The proportional hazards assumption was verified using Schoenfeld residual tests. Consequently, results from multivariable Cox regression should be interpreted as exploratory and hypothesis-generating rather than confirmatory. Statistical analyses were performed using SPSS Statistics version 26.0 (IBM Corp., Armonk, NY, USA) for all statistical tests and R version 4.3.1 (R Foundation for Statistical Computing, Vienna, Austria) for data visualization. All statistical tests were two-tailed, and *P* < 0.05 was considered statistically significant for univariable analyses, while multivariable results are presented with exact P-values without strict significance thresholds given their exploratory nature.

## Results

### Patient cohort and baseline characteristics

This study included 34 patients with histologically confirmed PCP who underwent GKRS at the Gamma Knife Center of Beijing Tiantan Hospital, Capital Medical University, from August 2002 to March 2025. The mean age was 41.71 ± 13.60 years, with a male-to-female ratio of 18:16 (Tables 1A and 1B). All patients had undergone prior surgical treatment before GKRS, with 20 patients (58.8%) receiving one surgery, 13 patients (38.2%) receiving two surgeries, and 1 patient (3.0%) receiving three surgeries. The mean interval between the last surgery and GKRS treatment was 21.87 ± 17.07 months (range: 3.08–64.10 months). The median tumor volume at the time of GKRS was 1.10 cm³ (range: 0.0625–7.9 cm³). None of the patients had received prior radiation therapy, chemotherapy, or targeted therapy before GKRS.

### Treatment

The median marginal dose was 12 Gy (range: 9–15 Gy), with a mean central dose of 23.98 ± 3.60 Gy. An average of 6 Gamma Knife shots were used, and the maximum mean dose to critical structures was 9.13 ± 0.65 Gy.

### Follow-up and tumor control

#### Follow-up duration

**Fig. 2:**
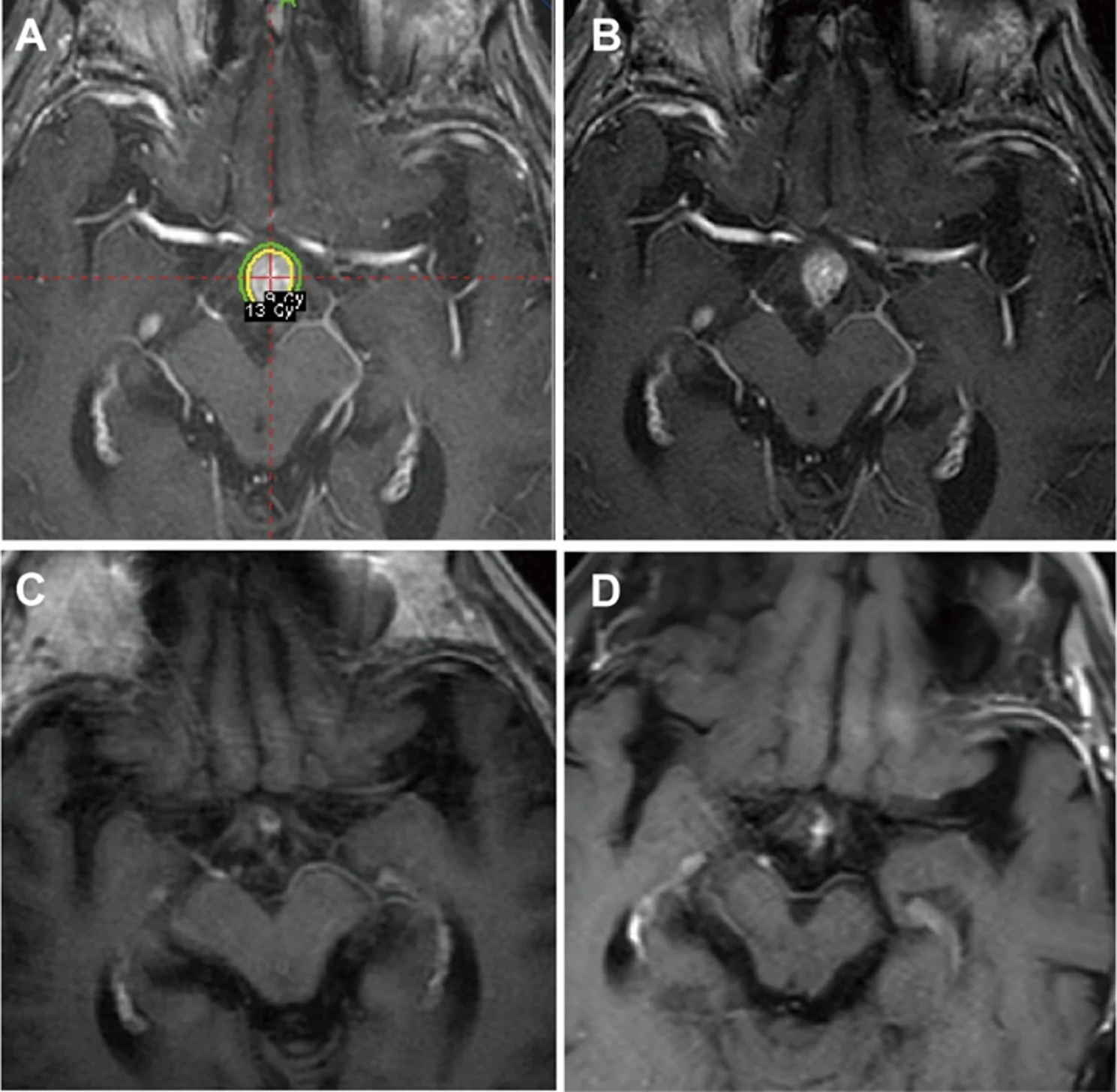
Representative case demonstrating tumor response to GKRS in a patient with PCP. A 51-year-old male patient with recurrent PCP underwent GKRS in 2012. (**A**) Treatment planning image showing the tumor target delineation with isodose lines (yellow contours) and dose distribution. The red line indicates the planning axis. (**B**) Pre-treatment T1-weighted contrast-enhanced MRI demonstrating a well-defined enhancing lesion in the sellar region. (**C**) One-year follow-up MRI (2013) showing partial response with significant tumor volume reduction. (**D**) Four-year follow-up MRI (2017) demonstrating sustained tumor control with further volume reduction and stable residual tumor. All images are T1-weighted with gadolinium enhancement, axial view

Clinical follow-up ranged from 7.36 to 274.56 months, with a mean follow-up of 78.48 months and median follow-up of 54.10 months. Radiological follow-up (*n* = 30, 4 patients did not undergo radiological follow-up) ranged from 9.64 to 145.68 months, with a mean follow-up of 42.84 months and median follow-up of 34.8 months. Figure 2 illustrates a representative case showing the tumor response to GKRS in a patient with PCP.

#### Imaging response rates

Based on best radiological follow-up results, complete response (CR) was achieved in 2 cases (5.88%), partial response (PR) in 8 cases (23.53%), stable disease (SD) in 14 cases (41.18%), and progressive disease (PD) in 10 cases (29.41%). The objective response rate (ORR) was 29.41%, and the disease control rate (DCR) was 70.59%.

#### Survival analysis

**Fig. 3:**
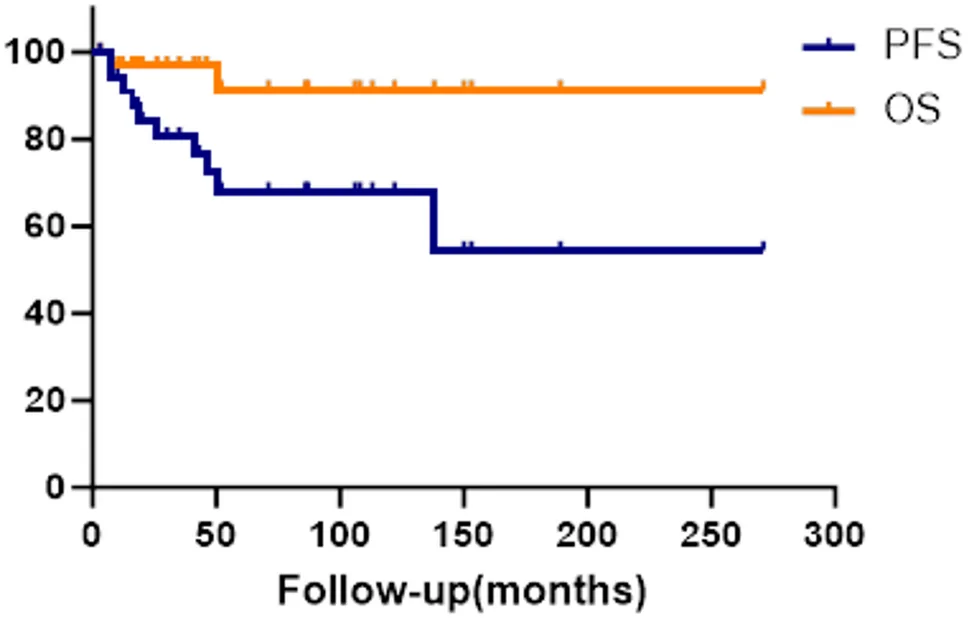
Progression-free survival and overall survival after GKRS. Kaplan–Meier curves showing PFS and OS in 34 patients with PCP treated with GKRS. The 1-, 3-, and 5-year PFS rates were 91.18%, 82.35%, and 73.53%, respectively. The overall survival rate was 94.12% at the last follow-up

Radiological progression occurred in 10 cases (29.41%), with a median time to progression of 22.5 months (range: 7-138 months). The 1-year, 3-year, and 5-year progression-free survival rates were 91.18%, 82.35%, and 73.53%, respectively (Fig. 3). Median PFS was not reached during the follow-up period. The overall survival rate was 94.12% (Fig. 3), with 2 patient deaths and no patients lost to follow-up.

**Fig. 4:**
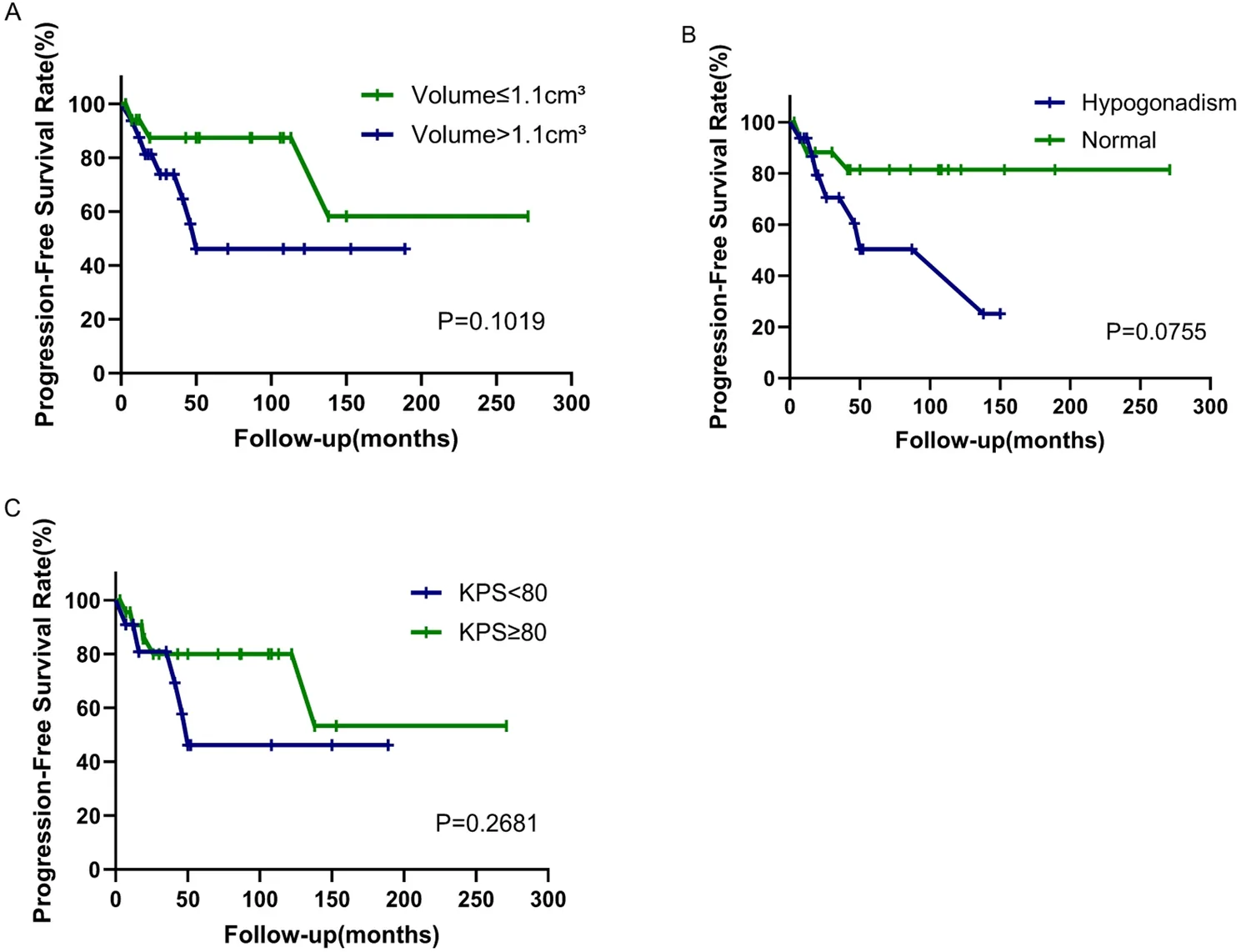
Stratified survival analysis of PCP patients treated with GKRS. (**A**) Stratification by tumor volume (≤1.1 cm³ vs >1.1 cm³) using the median as a pre-specified cut-off. (**B**) Stratification by preoperative gonadal function (normal vs hypofunction). (**C**) Stratification by preoperative KPS score (≥80 vs <80). All P-values were calculated using the log-rank test

Kaplan-Meier stratified survival analysis (Fig. 4) revealed that patients with tumor volume > 1.1 cm³ had a median progression-free survival of 50.0 months, while those with volume ≤ 1.1 cm³ did not reach median survival (*P* = 0.1019). Patients with hypogonadism had a median progression-free survival of 138.0 months, while those with normal function did not reach median survival (*P* = 0.0755). Patients with KPS < 80 had a median progression-free survival of 50.0 months, while those with KPS ≥ 80 did not reach median survival (*P* = 0.2681). Exploratory data-driven cut-point optimization is provided in Supplementary Figure S1.

### Exploratory analysis of factors associated with progression

The 34 patients were divided into a non-progression group (*n* = 24, 70.59%) and a progression group (*n* = 10, 29.41%). Univariable analysis showed that tumor volume (*P* = 0.116), preoperative hypogonadism (*P* = 0.134), and preoperative KPS score (*P* = 0.129) were associated with progression (*P* < 0.2). No multicollinearity existed between variables, and they were included in multivariable analysis (Table 2).

**Fig. 5:**
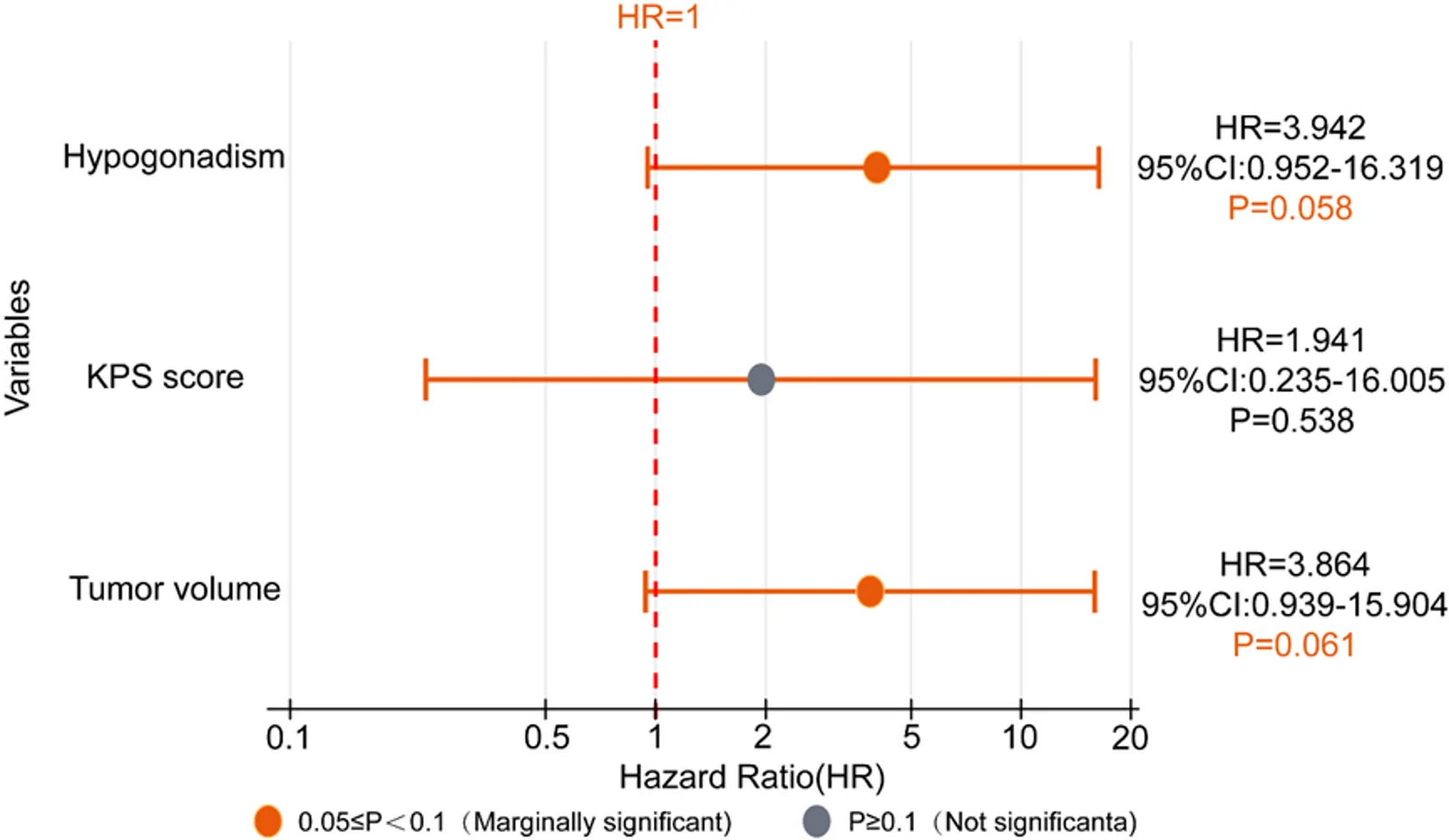
Forest plot represents the results of multivariable cox regression analysis

Given the limited number of progression events (*N* = 10) and three predictors in the multivariable model (EPV = 3.3), the following results should be interpreted as exploratory and hypothesis-generating rather than confirmatory (Table 3). Cox regression analysis suggested that tumor volume (HR = 3.864, 95% CI: 0.939–15.904, *P* = 0.061) and preoperative hypogonadism (HR = 3.942, 95% CI: 0.952–16.319, *P* = 0.058) may be associated with progression, though these trends did not reach statistical significance. KPS score showed no apparent association with progression (HR = 1.941, 95% CI: 0.235–16.005, *P* = 0.538). These findings suggest potential prognostic value of tumor volume and preoperative gonadal function, but require validation in larger cohorts with adequate statistical power. The forest plot (Fig. 5) visualizes the hazard ratios from this exploratory multivariable analysis.

### Treatment after progression

Among the 10 patients with progression, salvage treatment outcomes are summarized in Table 4. Seven (70%) received repeat GKRS treatment. Of these, 5 patients maintained tumor stability without further growth during subsequent follow-up; 1 patient experienced progression again after the second GKRS and subsequently received a third GKRS, with current follow-up showing disease stabilization; 1 patient died after receiving repeat GKRS at an external institution one year later. Three patients (30%) underwent repeat craniotomy, with 1 patient dying from complications after repeat surgery, while 2 patients showed good tumor control on postoperative follow-up assessment without new progression. The overall salvage treatment control rate was 80% (8/10 patients) for recurrent cases.

### Analysis of factors associated with visual deterioration in non-recurrent patients

To investigate potential radiation-induced optic neuropathy independent of tumor progression, we analyzed treatment parameters associated with visual deterioration in the non-progression group (*N* = 24). Among these patients, 4 (16.7%) experienced visual deterioration while 20 (83.3%) maintained stable or improved vision.

Comparative analysis revealed several notable findings (Table 5). Patients with visual deterioration had significantly lower isodose line selection (46.25 ± 2.50% vs. 50.0 ± 2.29%, *P* = 0.009), indicating steeper dose gradients and greater treatment complexity. Additionally, these patients demonstrated a trend toward larger tumor volumes (3.68 ± 3.24 cm³ vs. 1.06 ± 1.01 cm³, *P* = 0.075) and higher proportion of cystic tumors (75% vs. 30%, *P* = 0.108), although these differences did not reach statistical significance.

Importantly, no significant differences were observed in maximum dose to the optic pathway (8.9 ± 0.20 Gy vs. 8.97 ± 0.13 Gy, *P* = 0.226), marginal dose (11.50 ± 1.0 Gy vs. 11.25 ± 1.71 Gy, *P* = 0.633), or central dose (25.02 ± 3.35 Gy vs. 22.54 ± 3.62 Gy, *P* = 0.180) between groups. Similarly, tumor location (suprasellar involvement) and presence of calcification showed no significant association with visual deterioration.

Descriptive analysis showed that all 4 patients with visual deterioration had large tumor volumes (range: 0.51–7.90 cm³, median 2.72 cm³), with 3 of 4 (75%) being cystic tumors. These findings suggest that visual deterioration in non-recurrent patients is associated with tumor characteristics (size, consistency, anatomical complexity) and treatment planning challenges rather than excessive radiation dose to the optic apparatus.

### Functional outcomes and quality of life

Secondary outcome analysis revealed important functional outcomes following GKRS. Among all 34 patients, visual function remained stable in 20 patients (58.8%), deteriorated in 12 patients (35.3%), and improved in 2 patients (5.9%). Endocrine function was stable in 24 patients (70.6%), improved in 5 patients (14.7%), and deteriorated in 5 patients (14.7%) (Table 6). Based on retrospective functional assessment, quality of life showed mild impact in 21 patients (61.76%), moderate impact in 10 patients (29.41%), and severe impact in 3 patients (8.82%).

When stratified by treatment response, significant differences in visual changes were observed between the progression and non-progression groups (*P* = 0.002). The progression group had a significantly higher rate of visual deterioration compared to the non-progression group (80.0% vs. 16.7%), while 8.3% of patients in the non-progression group experienced visual improvement, with no visual improvement observed in the progression group. This pattern suggests an association between visual decline and tumor progression rather than radiation-related factors. Supporting this interpretation, among patients without tumor progression (*n* = 24), no significant association was found between visual outcome and maximum optic pathway dose, marginal dose, or central dose(Table 5).

Endocrine function changes showed no statistically significant difference between progression and non-progression groups (*P* = 0.114). Endocrine improvement occurred exclusively in the non-progression group (20.83%), with no patients in the progression group demonstrating endocrine improvement. The progression group had a numerically higher rate of endocrine deterioration compared to the non-progression group (30.0% vs. 8.3%), though this did not reach statistical significance.

These functional outcome patterns suggest that maintaining tumor control may be associated with better preservation of visual and endocrine function after GKRS. However, given the subjective nature of visual outcome assessment and heterogeneous follow-up protocols across different institutions over the 23-year study period, these associations require careful interpretation and warrant validation in prospective studies with standardized outcome assessment protocols.

### Fatal cases

Two patients died during follow-up. One was a 59-year-old female who presented with blurred vision and had a suprasellar cystic-solid tumor. The patient underwent near-total resection via right frontotemporal approach with poor postoperative condition, manifesting as somnolence, poor mental status, abdominal distension, and urinary frequency. Cystic recurrence occurred 7 months after GKRS treatment. One year later, she received repeat GKRS at an external institution but died 5 days post-treatment due to complications of heart failure and hypoxemia. The other case was a 51-year-old female who underwent 2 craniotomies for progression before GKRS, received another craniotomy 10 years later for visual decline, and subsequently died from complications. No serious adverse events were directly related to GKRS.

## Discussion

To our knowledge, this study represents one of the largest single-center Gamma Knife radiosurgery cohorts restricted to histologically confirmed papillary craniopharyngioma, with long-term follow-up. Although PCP account for approximately 20.9–28.8% of all craniopharyngiomas [26, 27], they differ fundamentally from ACP in driver mutations, biological behavior, and treatment response [8, 9, 28, 29]. In previously published GKRS series, the number of explicitly reported PCP cases ranged from 3 to 35 (Table 7), and these cases were typically analyzed together with ACP without subtype-specific outcome assessment [20, 30–33]. As a result, PCP-specific radiosurgical outcomes remain poorly characterized, despite the growing recognition that ACP and PCP represent biologically and clinically distinct entities. This study is the first to specifically report the results of GKRS for PCP. Through long-term follow-up observation of 34 PCP patients who received GKRS treatment postoperatively, with a median clinical follow-up time of 54.10 months, the results showed that PCP patients achieved a 5-year PFS rate of 73.53% and an overall survival rate of 94.12% after GKRS. These results are comparable to previous literature reports, where studies mixing ACPs and PCPs reported 5-year PFS rates ranging from 50% to 92.3% [15, 18, 20, 22, 34–36], with an average of 70.77%. However, this study provides long-term observation spanning 23 years of PCP natural history and GKRS efficacy. Although the 5-year PFS improvement was limited, the median time to progression was 22.5 months. Literature reviews show that the average postoperative progression rate for PCPs ranges from 22% to 27%, with a median time to progression of 15 months [23]. In a large study including 101 PCP patients, the tumor progression rate was approximately 12.87% during an average follow-up period of 38 months [37].

This study included a series of complex cases with multiple surgeries (41.18% of patients underwent ≥ 2 surgeries). Based on radiological follow-up results, 70.59% of patients achieved tumor control after GKRS. This favorable tumor control rate may be related to the BRAF V600E mutation in PCP, as BRAF V600E mutation can enhance radiosensitivity by activating the MAPK pathway [38, 39]. However, BRAF mutation status was not routinely measured in this cohort, and therefore this hypothesis regarding enhanced radiosensitivity remains speculative and requires validation in future studies with molecular characterization. Radiosurgical treatment of CP has also found that PCP show superior radiosensitivity compared to ACP [38]. In Mokry et al.‘s report of 23 patients receiving GKRS, all 6 patients with tumor progression were of the ACP [40]. Additionally, PCPs are predominantly solid in composition (52.94% solid component in this study), and compared to the cystic-dominant structure of ACPs, solid structures allow more tumor cells to receive higher doses of radiation, which may be the anatomical basis for higher tumor control rates [20, 21, 41].

Univariable analysis revealed that tumor volume, preoperative hypogonadism, and preoperative KPS score were associated with progression. In pre-specified exploratory multivariable Cox analysis, tumor volume and preoperative hypogonadism suggested potential associations with progression risk, though these did not reach statistical significance. We used median tumor volume (1.1 cm³) as a pre-specified cut-point for stratified analysis to avoid overfitting bias associated with data-driven threshold selection in small samples. Although data-driven cut-point optimization techniques such as ROC curve analysis exist, such approaches require validation in independent cohorts to avoid spurious associations, particularly when event numbers are limited. Our conservative approach using median dichotomization provides a reproducible, pre-specified threshold that does not capitalize on chance variation in our specific dataset. Larger tumor volume (> 1.1 cm³) showed a trend toward shorter progression-free survival (HR = 3.864, *P* = 0.061), with a median PFS of 50 months, while patients with smaller tumors did not reach median survival. This is consistent with previous study results - larger tumors progress faster [42, 43], and large tumors often require higher doses, but considering the need to reduce side effects, marginal and maximum doses of GKRS may be reduced, thereby affecting efficacy [41]. Literature indicates that volume thresholds range from 6 to 20 ml [20, 43, 44]. Larger tumors typically require more shots, and large-volume tumors often have more complex cystic-solid structures, which may affect dose coverage uniformity and increase progression risk [45]. Additionally, while multi-shot design attempts to improve target conformality, it may affect tumor cell killing effectiveness due to insufficient fractional dose splitting. However, our study found no significant correlation between peripheral dose and isodose curves with progression risk, which differs from some previous study results [46]. This difference may reflect the heterogeneity of dose regimens in different studies or limitations in statistical power, while our prescription doses were relatively uniform, mostly between 12 and 14 Gy, with insufficient range difference to show statistical significance. Preoperative hypogonadism as another important prognostic factor (HR = 3.942) may reflect the degree of tumor invasion of the hypothalamic-pituitary axis. Patients with functional impairment often indicate that tumors have already caused damage to critical anatomical structures, which may be related to tumor biological aggressiveness and treatment resistance.

This study demonstrates acceptable safety of GKRS, with no directly related serious adverse events. After GKRS, most patients (58.82%) maintained stable vision, 5.88% showed visual improvement, but 35.29% experienced visual deterioration. This visual complication rate is notably higher than some literature reports [30, 35, 47], warranting careful analysis of contributing factors. The key to understanding this elevated rate lies in stratified analysis by tumor response. Visual deterioration showed a striking disparity between groups: 80.0% of patients in the progression group versus only 16.7% in the non-progression group experienced visual worsening (*P* = 0.002). This strongly suggests that the majority of visual deterioration in our cohort was related to tumor progression rather than radiation-induced optic neuropathy. When tumor growth is excluded as a confounding factor, our radiation-related visual complication rate is consistent with published data. To specifically evaluate potential radiation-induced optic toxicity, we performed detailed comparative analysis within the non-progression group (*N* = 24). Contrary to the traditional dose-toxicity paradigm, we found no significant association between visual deterioration and radiation dose parameters, including maximum dose to the optic pathway (*P* = 0.226), marginal dose (*P* = 0.633), or central dose (*P* = 0.180). The median maximum optic pathway dose was nearly identical between groups (8.9 Gy vs. 8.97 Gy), both well within the established safety threshold of < 10 Gy for single-fraction radiosurgery [47]. Instead, our analysis revealed that visual deterioration was significantly associated with treatment complexity and tumor characteristics. Patients with visual worsening had significantly lower isodose line selection (46.25% vs. 50%, *P* = 0.009), which paradoxically suggests more conservative treatment planning rather than more aggressive dosing. Lower isodose lines are typically employed for tumors in close proximity to critical structures, creating steeper dose gradients to protect adjacent tissues. This finding indicates that visual deterioration occurred in anatomically challenging cases where tumors had intimate contact with the optic apparatus, necessitating complex dose optimization. Additionally, patients with visual deterioration demonstrated a strong trend toward larger tumor volumes (3.68 cm³ vs. 1.06 cm³, *P* = 0.075) and predominantly cystic consistency (75% vs. 30%, *P* = 0.108). These findings suggest that visual deterioration in non-recurrent patients is primarily driven by tumor characteristics and anatomical complexity rather than radiation dose. Larger tumors inherently have greater contact with optic structures, increasing baseline risk regardless of radiation dose. The 3.5-fold volume difference between groups indicates extensive pre-existing optic pathway involvement. Even with optimal dose planning maintaining optic pathway doses < 9 Gy, mechanical tumor effects and treatment-induced edema may precipitate visual decline in these anatomically compromised cases. The predominance of cystic tumors (75%) in the visual deterioration group is particularly relevant, as cystic craniopharyngiomas may exhibit acute expansion or peri-tumoral edema following radiosurgery [41], potentially causing optic pathway injury even without tumor progression. This mechanism likely explains the early visual deterioration (< 6 months) observed in half of our cases. Milano et al.‘s analysis established that single-fraction doses < 8 Gy to optic structures carry minimal risk (< 3%) of radiation-induced optic neuropathy, while doses of 10–15 Gy significantly increase risk [47]. Our median maximum optic pathway dose was 8.97 Gy in the non-progression group, with 91.2% of all patients receiving doses < 9 Gy. Critically, visual outcomes showed no correlation with optic pathway dose (*P* = 0.226), reinforcing that dose alone is not the primary determinant of visual toxicity in anatomically complex cases. The significantly lower isodose line selection in the visual deterioration group (46.25% vs. 50%, *P* = 0.009) likely reflects confounding by indication—these patients required more complex planning due to tumor proximity to optic structures, identifying them as inherently high-risk cases despite conservative dosing. For such anatomically challenging cases, alternative strategies warrant consideration: hypofractionated stereotactic radiotherapy to reduce acute edema risk, staged radiosurgery with interim assessment, or neoadjuvant BRAF/MEK inhibitor therapy to achieve tumor reduction before radiosurgery.

Regarding endocrine function, 15.63% of patients improved, 71.87% remained stable, and 12.5% deteriorated, indicating that GKRS has relatively limited impact on endocrine function. Previous studies showed that the incidence of endocrine dysfunction after GKRS of CP ranges from 16 to 27% [31, 46], and our results are at the low end of this range, indicating that for PCP patients who have already undergone surgery, GKRS is a relatively safe treatment option. Endocrine function improvement mainly occurred in the non-progression group, suggesting that successful tumor control helps endocrine function recovery. Quality of life assessment showed that most patients (65.62%) were only mildly affected, 28.13% moderately affected, and 6.25% severely affected. Niranjan et al. reported that approximately 70% of patients could maintain or improve quality of life after GKRS treatment of CP [36]. These data further support the overall safety and acceptability of GKRS as postoperative adjuvant treatment for PCP. However, our quality of life assessment methodology has important limitations (detailed in the Limitations section), including the absence of validated instruments and potential underestimation of cognitive and visual impacts that significantly affect patient well-being. Therefore, while our data suggest acceptable functional preservation in the majority of patients, prospective studies using validated quality of life instruments are essential to comprehensively characterize the patient experience following GKRS for PCP. Regarding treatment safety, careful patient selection and monitoring remain paramount. Two deaths occurred in our series, both in medically complex patients who underwent repeat treatment after progression. One patient died from heart failure and hypoxemia after repeat GKRS. Although direct causation cannot be established, this case emphasizes the need for careful evaluation of cardiopulmonary function and systemic status in PCP patients, particularly those with metabolic or cardiovascular complications secondary to hypothalamic-pituitary dysfunction, before considering repeat radiosurgery.

For recurrent cases, both repeat GKRS treatment (70%) and surgical intervention (30%) achieved good tumor control. 70% of recurrent patients received repeat GKRS, with 71.4% (5/7) achieving good tumor control. This result supports the feasibility of multiple GKRS treatments, and for selected recurrent cases, repeat GKRS may be a safe and effective salvage treatment strategy. These results are consistent with limited literature on repeat GKRS of CP [48]. However, it should be noted that repeat radiation therapy may increase the risk of radiation complications, including radiation necrosis, optic neuropathy, and hypothalamic-pituitary dysfunction. Nevertheless, for patients unsuitable for repeat surgery, repeat GKRS remains a worthwhile consideration, but special attention must be paid to the risk of radiation complications.

Particularly noteworthy is that BRAF gene mutations in PCPs in recent years have provided new possibilities for targeted therapy. Currently, targeted therapy using BRAF/MEK inhibitors has gradually become an effective treatment strategy for PCP [29, 49–51]. This study found that tumor volume is an important related factor for progression, so a very promising research direction is to explore sequential or combined strategies of BRAF targeted therapy with GKRS. BRAF inhibitors might first be used to shrink tumor volume and reduce complexity, followed by GKRS application to achieve more precise dose distribution and better tumor control. This multimodal treatment strategy may be particularly suitable for large-volume, complex, or location-sensitive PCP cases, potentially reducing progression rates and treatment-related complications. Therefore, future prospective clinical trials need to be designed to evaluate the safety, efficacy, and optimal timing of GKRS after BRAF inhibitor neoadjuvant treatment, providing evidence-based medical support for this innovative treatment modality.

## Limitations

For relatively rare tumors, standardized protocols within the same treatment center reduce confounding factors, providing relative consistency in data quality and treatment regimens. However, several important limitations must be acknowledged. First, methodological constraints limit the strength of our conclusions. The retrospective design may introduce selection bias and precluded systematic assessment protocols. The requirement for definitive pathological diagnosis resulted in a small sample size (*n* = 34) with limited statistical power. With only 10 progression events and 3 predictors, our multivariable Cox model was underpowered (events per variable = 3.3 vs. recommended ≥ 10); results should be considered exploratory and hypothesis-generating, and P-values approaching significance (0.058 and 0.061) may reflect insufficient power rather than true null effects. Radiological follow-up was incomplete in 4 patients, and the lack of prospective control groups precluded direct comparison with other treatment modalities (proton therapy, conventional fractionated radiotherapy).

Second, outcome assessment limitations affect data interpretation. Due to heterogeneous follow-up protocols across different institutions over 23 years, standardized neuroendocrine and visual assessments were not feasible. Quality of life was assessed using retrospective, investigator-defined categorization rather than validated instruments (SF-36, SF-12), limiting comparability with other studies. This approach may underestimate true patient burden, as deficits categorized as “moderate” (cognitive impairment requiring retirement, unilateral visual loss) can significantly impact independence and well-being. Clinician-documented outcomes may not fully capture patient-reported experiences. Although median follow-up reached 54.10 months, longer observation may be more valuable for this slow-growing tumor. Future prospective studies should employ validated patient-centered quality of life instruments, larger multicenter cohorts, and comparative treatment designs to confirm and extend these findings.

Third, the extended study period (2002–2025) introduces potential era effects that may confound outcome interpretation. During this timeframe, substantial technological advances occurred including upgrades from Leksell Gamma Knife Model C to Perfexion (2011) and subsequently to Icon with cone-beam CT guidance (2021), improvements in MRI resolution and treatment planning software capabilities, and evolution in dose prescription practices. These technological refinements may have improved treatment precision and outcomes in later-treated patients, creating heterogeneity that our analysis cannot fully account for. Treatment era and model upgrades may confound outcomes, potentially contributing to the observed variability in progression rates and complication profiles.

Fourth, in a benign tumor with long natural history and excellent overall survival, competing-risk considerations warrant acknowledgment. The detection and timing of “progression” and subsequent “intervention” decisions can be substantially influenced by surveillance imaging intensity, institutional follow-up protocols, and individual clinician thresholds for defining actionable tumor growth. Patients followed more intensively with frequent MRI surveillance may have progression detected earlier compared to those with less intensive monitoring, potentially leading to earlier salvage interventions that might not reflect true biological differences in tumor behavior. Similarly, varying clinician thresholds for what constitutes clinically significant progression (some may intervene at smaller volumetric changes while others adopt more conservative observation) introduce heterogeneity in outcome ascertainment. These factors may affect our progression-free survival estimates and comparisons with other series employing different surveillance strategies.

## Conclusion

This single-center retrospective study demonstrates that GKRS is an effective treatment option for patients with postoperative residual or recurrent PCP, providing favorable long-term tumor control rates and acceptable safety profiles, though comprehensive quality of life assessment using validated instruments is warranted in future prospective studies. Tumor volume and preoperative gonadal function status are important prognostic factors. These findings assist clinicians in risk stratification and treatment decision-making, providing important references for clinical decisions and follow-up strategies. Despite certain limitations, this study provides valuable evidence for the comprehensive management of PCPs, emphasizing the importance of multidisciplinary collaboration and individualized treatment. Future larger-scale, multicenter prospective studies are needed to validate these results and further optimize treatment strategies for PCPs.

**Table 1A:** Baseline characteristics of 32 patients with PCP treated with GKRS

Case	Sex	Year	Age at onset (y)	Surgical approach	Gross total resection	Calcification ^1^	Tumor consistency	Indication for GK^2^	Location	Recurrence at GK	Preoperative comorbidities
1	M	2024	61	TSA	No, NTR	No	Solid	Progression	Sellar	2	PHP, HTH, HH, HAD
2	F	2023	41	TSA	Yes	No	Solid	Progression	Sella, Clivus	2	DI
3	F	2024	41	TSA	Yes	No	Solid	Progression	Sella, Clivus	3	HAD, DI
4	F	2025	41	TSA	Yes	No	Solid	Progression	Sella, Clivus	4	HTH, DI
5	M	2021	31	TCA	Yes	Yes	Solid	Progression	Suprasellar	1	HH
6	M	2023	31	TCA	Yes	Yes	Solid	Progression	Suprasellar	2	HTH, HH
7	M	2023	34	TSA	Yes	No	Solid	Progression	Sellar	1	PHP, HTH, HH, HAD, DI
8	F	2023	61	TSA	Yes	No	Cystic	Progression	Sellar	1	HAD, DI
9	M	2017	48	TCA	Yes	Yes	Mixed	Progression	Sellar	2	HH, HAD, DI
10	M	2021	48	TCA	Yes	Yes	Mixed	Progression	Sellar	3	HH, HAD, DI
11	M	2022	48	TCA	Yes	Yes	Mixed	Progression	Sellar	4	HH, HAD, DI
12	F	2022	36	TSA	Yes	No	Cystic	Remnant	Suprasellar	1	HTH, DI
13	M	2021	29	TSA	Yes	No	Mixed	Progression	Sellar	1	HAD
14	F	2022	53	TCA	Yes	No	Solid	Progression	Sellar	1	HTH, HAD
15	F	2021	21	TCA	Yes	No	Mixed	Progression	Right frontal lobe, Third ventricle	2	HAD, DI
16	M	2021	48	TSA	Yes	No	Solid	Remnant	Suprasellar	0	HH, DI
17	M	2024	48	TSA	Yes	No	Solid	Progression	Suprasellar	1	PHP, HTH, HH, HAD, DI
18	F	2021	59	TCA	No, NTR	No	Mixed	Progression	Suprasellar	1	HTH, HH
19	M	2020	56	TSA	Yes	No	Solid	Progression	Suprasellar	1	HH, HAD, DI
20	M	2017	26	TCA	Yes	Yes	Mixed	Progression	Suprasellar	1	HH, HAD
21	F	2019	38	TSA	No, STR	No	Mixed	Progression	Intrasellar, Suprasellar	3	-
22	F	2018	51	TCA	Yes	No	Solid	Progression	Suprasellar	1	DI
23	M	2017	40	TCA	Yes	Yes	Solid	Remnant	Third ventricle	0	HH, HAD
24	F	2016	25	TCA	Yes	No	Solid	Progression	Third ventricle, Foramen of Monro	1	-
25	M	2016	26	TCA	Yes	Yes	Mixed	Progression	Intrasellar, Suprasella, Third ventricle	1	-
26	F	2016	44	TCA	Yes	No	Solid	Progression	Suprasella, Third ventricle	2	HAD, DI
27	M	2015	58	TCA	Yes	No	Solid	Progression	Suprasellar	1	HTH, DI
28	F	2015	49	TCA	No, PR	No	Mixed	Remnant	Suprasellar	1	DI
29	M	2013	38	TCA	Yes	Yes	Mixed	Progression	Sellar	2	HH
30	F	2012	38	TCA	Yes	Yes	Mixed	Remnant	Suprasellar	0	HAD
31	M	2012	51	TCA	Yes	Yes	Solid	Remnant	Suprasellar, Third ventricle	0	HH
32	M	2002	14	TCA	Yes	No	Cystic	Progression	Suprasellar	1	DI
33	F	2011	41	TCA	No, NTR	No	Mixed	Remnant	Sellar	1	PHP, HTH, HH, HAD, DI
34	F	2009	58	TCA	No, STR	No	Mixed	Remnant	Sellar	0	DI

F, female, M, male; TSA: Transsphenoidal approach; TCA: Transcranial approach; NTR: Near-total resection; STR: Subtotal resection; PR: Partial resection; GK: Gamma Knife; PHP: Panhypopituitarism; HTH: Hypothyroidism; HH: Hypogonadism; HAD: Hypoadrenalism; DI: Diabetes insipidus

1: Calcification features on initial radiological examination; 2: Progression: salvage therapy for progression, Remnant: adjuvant therapy for residual tumor remaining after surgery.

Recurrence at GK: Number of tumor recurrences prior to GKRS treatment (treatment indication, not outcome)

**Table 1B:** Treatment parameters and outcomes (continued from Table 1A)

Case	Age at GK (y)	Tumor volume (cm^3^) ^†^	GK shots	Central dose (Gy)	Margin dose (Gy)	Isodose line (%)	Maximum dose to critical structure (Gy)	Visual outcome after GKRS^‡^	Endocrine outcome after GKRS^§^	Progression	PFS(m)
1	63	0.33	3	18	9	50	Optic chiasm < 9	Stable	Stable	No	12
2	43	1.19	12	24	12	50	Brainstem < 12	Stable	Stable	Yes	12
3	44	0.75	8	20	11	55	Brainstem < 11	Worsened	Stable	Yes	7
4	44	1.05	13	22	11	50	Optic chiasm < 9	Stable	Stable	No	1
5	33	3.17	7	24	12	50	Optic nerve<9	Stable	Stable	Yes	26
6	35	1.37	8	20	10	50	Optic nerve < 9	Stable	Stable	No	20
7	35	0.47	3	24	12	50	Optic nerve < 9	Improved	Stable	No	19
8	63	1.24	6	26.7	12	45	Optic chiasm < 9	Stable	Stable	No	18
9	50	2.04	5	26.7	12	45	Optic nerve < 9	Worsened	Stable	Yes	46
10	54	7.01	11	20	10	50	Optic chiasm < 9	Worsened	Stable	Yes	7
11	54	4.83	6	20	9	45	Optic nerve < 9	Stable	Stable	No	33
12	38	1.52	8	18	9	50	Optic chiasm < 9	Worsened	Stable	Yes	41
13	31	0.13	4	20	10	50	Optic chiasm < 9	Stable	Stable	No	44
14	55	1.92	6	20	10	50	Optic nerve < 9	Worsened	Improved	No	30
15	28	0.51	2	30	15	50	-	Worsened	Stable	No	50
16	49	0.20	5	24	12	50	Optic chiasm<9	Worsened	Stable	Yes	19
17	52	1.07	5	22	11	50	Optic nerve < 9	Stable	Improved	No	11
18	60	2.59	11	18	9	50	Optic nerve < 9	Worsened	Deteriorated	Yes	**7** ^**¶**^
19	57	0.09	3	28	14	50	Optic nerve, Optic chiasm < 9	Stable	Deteriorated	No	52
20	26	0.06	3	20	11	55	Optic nerve < 9, Optic chiasm < 9	Improved	Improved	No	88
21	41	4.40	7	28.9	13	45	Optic nerve < 9	Worsened	Stable	No	62
22	54	0.60	3	26	13	50	Optic chiasm < 9	Stable	Improved	No	87
23	42	0.86	7	24	12	50	Optic nerve, Optic chiasm < 9	Stable	Improved	No	88
24	26	1.37	6	26.7	12	45	Optic chiasm < 9	Stable	Stable	No	108
25	27	1.10	6	24	12	50	Optic chiasm < 9	Stable	Deteriorated	No	108
26	46	0.77	12	24.4	11	45	Optic nerve < 9	Stable	Stable	No	106
27	59	1.63	7	26.7	12	45	Optic Pathway < 9	Stable	Stable	No	123
28	49	1.06	4	26	13	50	-	Stable	Stable	No	113
29	39	1.00	4	28.9	13	45	Optic nerve < 9	Worsened	Deteriorated	Yes	138
30	39	1.50	11	28	14	50	Optic nerve < 8.4	Stable	Stable	No	153
31	52	1.10	4	28.9	13	45	Optic nerve < 9	Stable	Stable	No	150
32	16	0.58	3	21.82	12	55	Optic nerve < 9	Stable	Stable	No	272
33	43	3.50	3	26.67	12	45	Optic nerve < 9.3	Worsened	Deteriorated	Yes	50^**¶**^
34	58	7.90	6	28.89	13	45	Optic nerve < 8.6	Worsened	Stable	No	189

†: Volume measured on pre- Gamma Knife MRI; ‡: Visual outcome was based on patient-reported perception of vision compared to baseline: improved, stable, or worsened; §: Endocrine outcome was inferred from hormone replacement adjustments across follow-ups. Deterioration was defined as initiation of new hormone therapy, ≥ 50% increase in existing dosages sustained for ≥ 3 months, or addition of new medications for inadequate control; unchanged dosage was considered stable; and decreased dosage with effective symptom control was interpreted as improved. ¶: Death occurred after progression of the tumor

**Table 2:** Univariable analyses for tumor progression

Variables	Non-progression (*N* = 24)	Progression (*N* = 10)	t/Z/χ²	*P* value
Preoperative Variables				
Age at onset (y)	41.71 ± 13.60	43.10 ± 7.90	-0.373	0.712
Male n/ (%)	13(54.17)	5(50.0)	0.049	1.000
Calcification n/ (%)	7(29.17)	4(40.0)	0.379	0.692
Tumor consistency n/ (%)			0.573	0.751
Solid	13(54.17)	4(40.0)		
Mixed	9(37.5)	5(50.0)		
Cystic	2(8.3)	1(10.0)		
Preoperative comorbidities n/ (%)				
Diabetes insipidus	13(54.17)	7(70.0)	0.731	0.467
Panhypopituitarism	3(12.5)	1(10.0)	0.043	1.000
Hypogonadism	9(37.5)	7(70.0)	2.993	**0.134**
Hypothyroidism	7(29.17)	3(30.0)	0.002	1.000
Hypoadrenalism	13(54.17)	4(40.0)	0.567	0.708
KPS score	80(75,90)	75(70,80)	-1.558	**0.129**
GTR (Yes/No) n/ (%)			0.054	1.000
Yes (%)	20(83.3)	8(80.0)		
No (%)	4(16.67)	2(20.0)		
Reason for GKRS			0.330	0.666
Residual (%)	5(20.83)	3(30.0)		
Recurrent (%)	19(79.17)	7(70.0)		
Progression at GK	1(1,2)	1.5(1,2)	-1.018	0.334
Surgery-to-Recurrence Interval(m)	20.00(9.50,36.50)	16.00(9.00,28.50)	-0.434	0.684
**Operative Variables**				
Age at GKRS(y)	43.88 ± 13.44	45.30 ± 8.06	-0.381	0.706
Tumor volume(cm^3^)	1.07(0.55,1.44)	1.78(1.00,3.17)	-1.588	**0.116**
Central dose (Gy)	24.38 ± 3.58	23.03 ± 3.84	0.979	0.335
Margin dose (Gy)	12.00(11.00,13.00)	12.00(10.00,12.00)	-1.031	0.313
Isodose level (%)	50.00(45.00,50.00)	50.00(45.00,50.00)	-0.216	0.962

GTR: Gross total resection. Bold values indicate *P* < 0.2, which were considered for inclusion in multivariable analysis

**Table 3:** Univariable and exploratory multivariable cox proportional hazards regression analysis of factors associated with PFS

Variables	Univariable	Multivariable Cox regression analysis		
	analysis *P*	HR	95% CI	*P* Value
**Preoperative Variables**				
Hypogonadism	**0.134**	3.942	0.952–16.319	**0.058**
KPS score	**0.129**	1.941	0.235–16.005	0.538
**Operative Variables**				
Tumor volume(cm^3^)	**0.116**	3.864	0.939–15.904	**0.061**

HR, hazard ratio; CI, confidence interval; KPS, Karnofsky Performance Status

**Table 4:** Salvage treatment outcomes in patients with progression (*N* = 10)

Salvage Modality	Number Treated	Tumor Control at Last Follow-up	Complications	Deaths
Repeat GKRS	7(70%)	6/7(85.7%)*	None reported	1(14.3%)
Repeat Surgery	3(30%)	2/3(66.7%)	1 perioperative death	1(33.3%)
**Overall**	10(100%)	8/10(80%)	1 perioperative death	2(20.0%)

*:Includes 5 patients with stable disease after initial repeat GKRS and 1 patient who achieved disease stabilization after third GKRS following second progression

**Table 5:** Comparison of treatment parameters in non-progression(*N* = 24) group by visual outcome

Variables	No Visual Worsening (*N* = 20)	Visual Worsening (*N* = 4)	Z/χ²	*P* value
Calcification n/ (%)	7(35%)	0	1.976	0.283
Tumor consistency n/ (%)			2.954	0.108
Solid	12(60%)	1(25%)		
Mixed	2(10%)	0		
Cystic	6(30%)	3(75%)		
Suprasellar location n/ (%)	12(60%)	1(25%)	1.645	0.300
Tumor volume(cm^3^)	1.06 ± 1.01	3.68 ± 3.24	-1.782	**0.075**
Central dose (Gy)	22.54 ± 3.62	25.02 ± 3.35	-1.342	0.180
Margin dose (Gy)	11.25 ± 1.71	11.50 ± 1.0	-0.477	0.633
Isodose level (%)	50 ± 2.29	46.25 ± 2.50	-2.622	**0.009**
Maximum dose to optic pathway(Gy)	8.97 ± 0.13	8.9 ± 0.20	-1.211	0.226

Bold values indicate *P* < 0.1

**Table 6:** Comparison of functional outcomes between non-progression and progression groups

Postoperative Variables	Non-progression (*N* = 24)	Progression (*N* = 10)	χ²	*P* value
Visual outcome n/ (%)			12.486	**0.002**
Improved	2(8.33)	0(0)		
Stable	18(75.0)	2(20)		
Worsened	4(16.67)	8(80)		
Endocrine outcome n/ (%)			4.337	**0.114**
Improved	5(20.83)	0(0)		
Stable	17(70.83)	7(70)		
Deteriorated	2(8.3)	3(30)		

**Table 7:** Summary on GKRS treatment for CP with pathological classification

Author &Year	No. of PCP patients	Total patients	Mean margin dose(Gy)	Mean Volume (cm^3^)	Tumor Control Rate %
Mokry 1999[40]	4(17.4%)	23	8–10	7	74
Kobayashi et al. 2012[43]	35(32.7%)^1^	107	11.5	3.5	80
Samanci et al. 2023[21]	5(20.8%)	24	20	2.4	77.3
Gupta et al. 2024[15]	3(8.57%)	35	12	3.25	91.4

1: squamous cell type by univariable difference analysis, *p* = 0.036

## Supplementary Information

Below is the link to the electronic supplementary material.

Supplementary Material 1

## Data Availability

The datasets analyzed during the current study are available from the corresponding author on reasonable request, subject to patient privacy protection and institutional approval.

## Abbreviations

ACP: Adamantinomatous craniopharyngioma
CP: Craniopharyngioma
PCP: Papillary craniopharyngioma
GKRS: Gamma Knife radiosurgery
SRS: Stereotactic radiosurgery
TCA: Transcranial approach
TSA: Transsphenoidal approach
GTR: Gross total resection
NTR: Near-total resection
STR: Subtotal resection
KPS: Karnofsky Performance Status
QoL: Quality of life
DI: Diabetes insipidus
HAD: Hypoadrenalism
HH: Hypogonadism
HTH: Hypothyroidism
PHP: Panhypopituitarism
CBCT: Cone-beam computed tomography
MRI: Magnetic resonance imaging
CR: Complete response
DCR: Disease control rate
ORR: Objective response rate
PD: Progressive disease
PR: Partial response
SD: Stable disease
OS: Overall survival
PFS: Progression-free survival
AUC: Area under the curve
CI: Confidence interval
EPV: Events per variable
HR: Hazard ratio
IQR: Interquartile range
ROC: Receiver operating characteristic
