# Supplementary material for: Gamma Knife treatment of papillary craniopharyngioma: a single-center study of tumor control and predictors of progression
Source: Radiat Oncol. 2026 Jul 8;21:132. doi: 10.1186/s13014-026-02875-0 (PMC13632162; doi:10.1186/s13014-026-02875-0)
Supplement: Supplementary file 1 [file 13014_2026_2875_MOESM1_ESM.docx]

Figure S1.Exploratory analysis using ROC-derived optimal tumor volume cutoff (≤1.978 cm³ vs >1.978 cm³). The cut-off was determined by maximizing the Youden index from receiver operating characteristic curve analysis for progression prediction (AUC = 0.675, sensitivity 50%, specificity 87.5%).
